# The Impact of Medium Chain and Polyunsaturated ω-3-Fatty Acids on Amyloid-β Deposition, Oxidative Stress and Metabolic Dysfunction Associated with Alzheimer’s Disease

**DOI:** 10.3390/antiox10121991

**Published:** 2021-12-14

**Authors:** Janine Mett

**Affiliations:** Biosciences Zoology/Physiology-Neurobiology, ZHMB (Center of Human and Molecular Biology), Faculty NT—Natural Science and Technology, Saarland University, D-66123 Saarbrücken, Germany; janine.mett@uni-saarland.de

**Keywords:** medium chain fatty acids, polyunsaturated fatty acids, decanoic acid, eicosapentaenoic acid, docosahexaenoic acid, antioxidants, oxidative stress, energy metabolism, amyloid-β, Alzheimer’s disease

## Abstract

Alzheimer’s disease (AD), the most common cause of dementia in the elderly population, is closely linked to a dysregulated cerebral lipid homeostasis and particular changes in brain fatty acid (FA) composition. The abnormal extracellular accumulation and deposition of the peptide amyloid-β (Aβ) is considered as an early toxic event in AD pathogenesis, which initiates a series of events leading to neuronal dysfunction and death. These include the induction of neuroinflammation and oxidative stress, the disruption of calcium homeostasis and membrane integrity, an impairment of cerebral energy metabolism, as well as synaptic and mitochondrial dysfunction. Dietary medium chain fatty acids (MCFAs) and polyunsaturated ω-3-fatty acids (ω-3-PUFAs) seem to be valuable for disease modification. Both classes of FAs have neuronal health-promoting and cognition-enhancing properties and might be of benefit for patients suffering from mild cognitive impairment (MCI) and AD. This review summarizes the current knowledge about the molecular mechanisms by which MCFAs and ω-3-PUFAs reduce the cerebral Aβ deposition, improve brain energy metabolism, and lessen oxidative stress levels.

## 1. Fatty Acids

**Figure 1:**
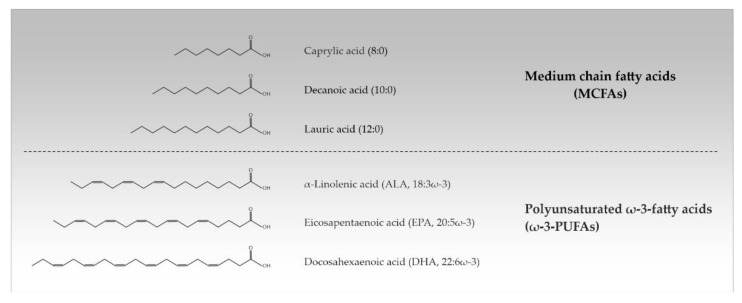
Chemical structures of the most important medium chain and polyunsaturated ω-3-fatty acids (MCFAs, ω-3-PUFAs).

Approximately 50% of the human brain’s dry weight consists of lipids, making it the second most lipid-rich organ in the body after adipose tissue. The diversity of lipids is mainly based on the variation of fatty acids (FAs) which can differ in the length and saturation degree of their hydrocarbon chain. This determines their physical properties such as their melting point [1]. FAs from 2 to 30 carbons or more naturally occur, but the most common ones contain between 12 and 24 carbon atoms. Depending on their acyl chain length, FAs are divided into short chain FAs (SCFAs) (≤6 carbon atoms), medium chain FAs (MCFAs) (8–12 carbon atoms), long chain FAs (LCFAs) (14–18 carbon atoms), and very long chain FAs (VLCFAs) (≥20 carbon atoms). Additionally, a distinction is made between saturated FAs (SFAs) (no double bonds), monounsaturated FAs (one double bond), and polyunsaturated FAs (≥2 double bonds). PUFAs are referred to as ω-3- and ω-6-PUFAs when the first double bond is localized between the 3rd/4th and the 6th/7th carbon atom from the terminal methyl group in their structure, respectively [2,3] (Table 1, Figure 1).

The term ‘essential’ is applied only to those FAs that are required for good health and cannot be completely synthesized in the human body. With α-linolenic acid (ALA, 18:3ω-3) indicating a length of 18 carbon atoms and 3 double bonds in ω-3-position) and linoleic acid (LA, 18:2ω-6) there are only two FAs that are known to be essential for humans; they must be supplied with diet. The oils of safflower, sunflower, and grapeseed have a high content of LA, rich ALA is found in linseed oil. The human body exhibits a limited ability to elongate ALA and LA to longer-chained PUFAs. Administration of LA into the body enables the formation of longer ω-6 FAs, while ALA is converted into longer-chained ω-3 FAs such as eicosapentaenoic acid (EPA; 20:5ω-3) and docosahexaenoic acid (DHA; 22:6ω-3). EPA (20:5ω-3) and DHA (22:6ω-3) are synthesized in large amounts by marine algae and can be dietary supplemented in the form of fish and marine oils [2,3]. Unique among dietary fats also is coconut oil with 62–70% MCFAs of the SFA portion (about 92%) [4]. PUFAs and MCFAs that are ingested with food can change the cerebral FA composition upon passing the blood-brain barrier [5,6,7,8,9,10,11].

Supplemented FAs are rapidly incorporated into the phospholipids of cellular membranes affecting the membrane structure, microdomain organization, and fluidity. Saturated longer-chained FAs are known to increase the gel-to-fluid phase transition temperature (melting temperature, Tm) of phospholipid bilayers and hence reduce membrane fluidity. In contrast, shorter-chained and unsaturated FAs have the opposite effect. Such alterations in the biophysical properties of cellular membranes can affect the trafficking of cellular constituents, membrane protein function, and signal propagation [12,13]. Besides their role as essential components of cellular membranes, FAs have a wide range of further functions within the central nervous system. They represent energy sources, signaling molecules, and are known to influence gene transcription, neuronal activity, as well as neuro-inflammatory and apoptotic processes [1,3]. There are three families of proteins sensing and transducing the signals of free FAs (FFAs) in neuronal cells: 1. G-protein-coupled free fatty acid receptors (FFARs) in the plasma membrane, 2. cytosolic fatty acid binding proteins (FABPs) involved in FA transport, and 3. nuclear peroxisome proliferator-activated receptors (PPARs) acting as transcription factors [12,14].

The role of FAs in human health and their potential in the prevention and/or treatment of various diseases has become more and more apparent. In general, an excess of saturated LCFAs and VLCFAs is considered as unhealthy whereas ω-3-PUFAs are associated with health benefits. A series of neurological disorders is connected to a dysregulated cerebral lipid homeostasis and particular changes in brain FA composition. These include depression, schizophrenia, Parkinson’s disease (PD), and Alzheimer’s disease (AD). Epidemiological studies and experimental research indicate that nutritional therapies that are based on FAs can be of benefit to several neurodegenerative and neurological diseases [3]. As described below, the dietary supplementation of MCFAs and ω-3-PUFAs seems to be advantageous in mild cognitive impairment (MCI) and the early stages of AD. Moreover, in the following chapters of this article, I will summarize the current knowledge about the impact of these special FAs on the molecular mechanisms that are associated with AD focusing on amyloid-β (Aβ) deposition, oxidative stress, and neuronal energy metabolism dysfunction.

## 2. Alzheimer’s Disease

**Figure 2:**
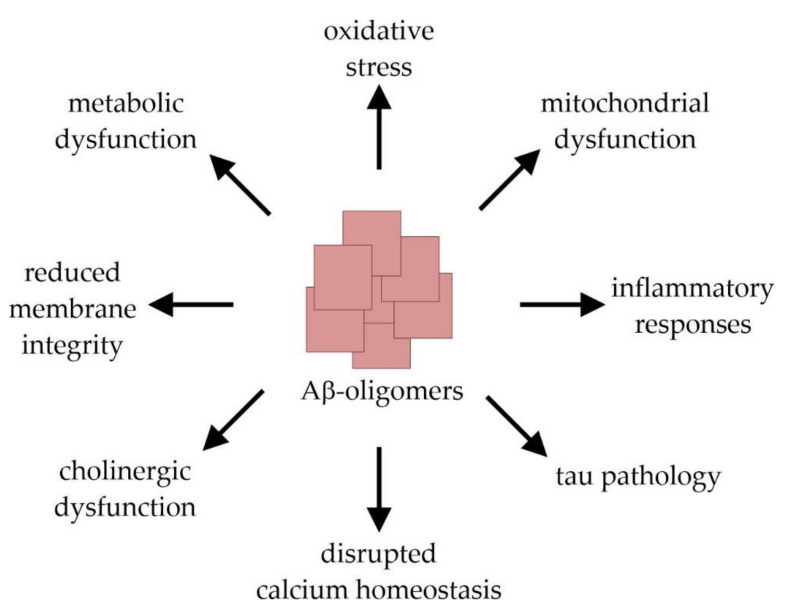
Molecular mechanisms of amyloid-β (Aβ)-induced toxicity leading to synaptic dysfunction and neuronal cell damage.

AD is the most common cause of dementia in the elderly population and affects tens of millions of people worldwide. Clinically, AD is characterized by a progressive decline in cognitive brain functions that finally result in a total loss of memory and personality [9,15]. This clinical picture is caused by an extensive loss of neurons and synapses which predominantly occurs in the hippocampal and cortical regions of the brain. Macroscopically, AD is characterized by a symmetric pattern of cortical atrophy mainly affecting the medial temporal lobes and relatively sparing the primary motor, sensory, and visual cortices. The characteristic neuropathological hallmarks of the disease further include the presence of extracellular amyloid plaques and intracellular neurofibrillary tangles (NFTs) [16]. The latter are mainly composed of the microtubuli-associated protein tau in a misfolded and abnormally hyperphosphorylated state [17,18]. The tau pathology follows a stereotypical spatiotemporal progression that strongly correlates with the severity of dementia [19,20]. The formation of amyloid plaques results from the abnormal extracellular accumulation and deposition of the peptide Aβ, which is considered as an early toxic event in AD pathogenesis triggering the disease process [21,22]. The 38–43 amino acid (aa) long, hydrophobic Aβ-monomers are physiologically derived from the amyloid precursor protein (APP) after its sequential cleavage by two proteases, β- and γ-secretase [23,24,25,26]. Due to its higher tendency to aggregate compared to the more prevalent Aβ40 (indicating 40 aa), Aβ42 represents the principal Aβ-species that is deposited in AD-brain tissue [27,28,29]. The aggregation process of Aβ-monomers involves distinct intermediates: dimers and trimers, oligomers (fibrillar, non-fibrillar and high molecular weight oligomers), protofibrils, and fibrils [22]. Importantly, it is not the large amyloid plaques, but rather the small Aβ-oligomers that elicit neuro- and synaptotoxicity. By interrupting the functional integrity of cellular membranes, they can initiate a series of events leading to cellular dysfunction and death [30,31,32]. These include the induction of tau pathology, neuroinflammation and oxidative stress, the disruption of calcium homeostasis and membrane integrity, an impairment of cerebral energy metabolism, as well as synaptic dysfunction and mitochondrial damage [22] (Figure 2).

## 3. ω-3-PUFAs and MCFAs in Mild Cognitive Impairment and Alzheimer’s Disease

Besides arachidonic acid (20:4ω-6), DHA (22:6ω-3) represents the most abundant PUFA that is incorporated in brain tissue and accounts for about 7–8% of the total cerebral FAs weight [33,34,35]. Several studies reported a declined EPA (20:5ω-3)- and DHA (22:6ω-3)-content in post-mortem AD brain tissue and in serum/plasma samples of persons that were suffering from AD [36,37,38,39]. Owing to their double bounds, PUFAs are very susceptible to lipid-peroxidation. The level of PUFA oxidation products is elevated in AD brain tissue, pointing to an enhanced oxidative damage of these FAs under the pathological conditions [40,41,42]. Conversely, an increased dietary uptake and higher plasma/serum levels of EPA (20:5ω-3) and DHA (22:6ω-3) were found to be associated with a reduced risk for developing AD in several trials [43,44,45,46]. Especially an increased ω-3/ω-6-ratio seems to negatively correlate with cognitive decline and the incidence of AD [47]. Persons suffering from MCI or mild dementia due to AD show improved cognitive performances after the dietary supplementation of EPA (20:5ω-3) and DHA (22:6ω-3) [48,49,50]. As illustrated by using AD animal models, these effects could be based on a negative correlation between the cerebral Aβ-content/Aβ-plaque formation and the uptake of ω-3-PUFAs [51,52,53,54,55]. However, it should be noted that other authors failed to find any association between the ω-3 PUFA status and AD-risk or disease progression [56,57,58,59]. 

Like PUFAs, MCFAs that are nutritionally administered as medium chain triglycerides (MCTs) or coconut oil seem to ameliorate the cognitive functions of patients that are suffering from MCI and early stages of AD [60,61,62,63,64,65]. Improved novel object recognition memory and social recognition has been reported for aged Wistar rats after the dietary supplementation of MCTs as well [66]. As described in detail below, MCFAs are metabolized within liver mitochondria resulting in the generation of β-hydroxybutyrate (BHB), acetoacetate, and acetone. Besides glucose, brain cells can use these hepatically derived major ketone bodies as fuel. Thus, the beneficial effects of MCFAs are often attributed to an improvement of cerebral energy metabolism [4,11,67]. However, some studies have challenged the central role of ketones, since MCFAs can promote neuronal health and improve cognition independent of ketone levels [11,66,68].

## 4. The Impact of ω-3-PUFAs and MCFAs on Aβ Deposition

**Figure 3:**
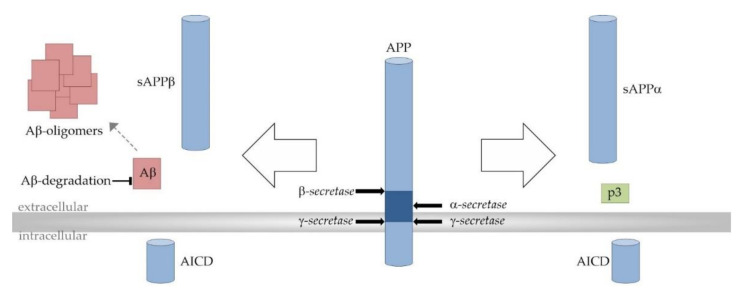
Proteolytic processing of the amyloid precursor protein (APP). Amyloidogenic APP processing pathway (left): Amyloidogenic APP processing is initiated by β-secretase (β-site APP cleaving enzyme 1, BACE1)-dependent cleavage of APP within its extracellular domain, generating the soluble β-secreted APP (sAPPβ) and the C-terminal membrane-bound fragment C99/β-CTF (APP–β-carboxyl terminal fragment) as intermediate product (not shown). C99/β-CTF is further cleaved by the γ-secretase complex resulting in the release of amyloid-β (Aβ) peptides (red) into the extracellular space. The aggregation of Aβ involves distinct intermediates and finally leads to the generation of larger Aβ aggregates and amyloid plaques (red). Total cerebral Aβ levels and amyloid pathology are also determined by Aβ elimination involving transport processes and its enzymatic degradation. Non-amyloidogenic APP processing pathway (right): In the non-amyloidogenic pathway APP is first processed by the α-secretases (A Disintegrin and metalloproteinase domain-containing proteins 10 and 17, ADAM10 and ADAM17). α-secretase-dependent APP cleavage generates soluble α-secreted APP (sAPPα) and the membrane spanning fragment C83/α-CTF (APP–α-carboxyl terminal fragment) as intermediate (not shown). C83/α-CTF is further cleaved by the γ-secretase complex to generate the non-toxic peptide p3 (green). Since the α-secretase cleavage site in APP is located within the Aβ domain (dark blue), the formation of Aβ peptides is precluded in the non-amyloidogenic APP processing pathway. In both APP processing pathways, the transcriptionally active APP intracellular domain (AICD) is released into the cytosol.

As already mentioned, Aβ peptides are derived from the precursor protein APP through the successive actions of β- and γ-secretase. Due to multiple-site cleavages that are performed by γ-secretase, Aβ peptides can vary in length with Aβ38, Aβ40, and the most neurotoxic, Aβ42, as the main products. Alternatively, APP can be processed in the predominant α- and γ-secretase-dependent non-amyloidogenic cleavage cascade completely precluding the formation of Aβ peptides. APP processing strongly depends on the surrounding lipid bilayer since APP and all secretases are transmembrane proteins. γ-secretase-dependent APP cleavage even takes place within the hydrophobic membrane environment [69] (Figure 3).

DHA (22:6ω-3) reduces Aβ generation by shifting the proportion of amyloidogenic and non-amyloidogenic APP processing. It inhibits the enzymatic activities of β- and γ-secretase directly and by affecting the intracellular trafficking of the β-secretase BACE-1 (β-site APP-cleaving enzyme 1) and the association of the γ-secretase component presenilin 1 (PS1) with lipid raft membrane microdomains. Additionally, DHA (22:6ω-3) and other FAs with four or more double bonds, such as EPA (20:5ω-3), were found to stimulate α-secretase activity and thus non-amyloidogenic APP processing [70,71,72]. Total cerebral Aβ levels and amyloid pathology are not only determined by Aβ generation, but also by Aβ elimination involving a variety of transport processes and its enzymatic degradation [26,73]. Insulin-degrading enzyme (IDE), a zinc-requiring metalloprotease, is a key enzyme that is involved in Aβ degradation in brain tissue. Cerebral Aβ accumulation is strongly increased in IDE-deficient mice, while it is reduced in the brain tissue of mice overexpressing the enzyme [74,75]. We observed a strongly enhanced IDE-dependent Aβ40 degradation in neuroblastoma cells that were supplemented with DHA (22:6ω-3) or EPA (20:5ω-3) [76]. A stimulating effect on the microglial Aβ42 phagocytosis and interstitial Aβ clearance has been also reported for ω-3-PUFAs [77,78]. Accordingly, the beneficial Aβ-lowering effects of ω-3-PUFAs are not only based on a reduction of Aβ generation but also on a stimulation of its elimination. DHA (22:6ω-3) seems to additionally inhibit the in vitro aggregation of Aβ and to resist the Aβ-induced toxicity in neuroblastoma cells [79,80]. 

Compared to ω-3-PUFAs, less is known about the relevance of MCFAs on the anabolism and catabolism of Aβ peptides. FA acyl chain length has been shown to influence γ-secretase activity in a cell-free system indicating direct effects on the enzyme’s catalytic activity. In this study FA acyl chain length in the range of 14 to 20 carbon atoms positively correlated with γ-secretase activity. Interestingly, the ratio of produced Aβ42/Aβ40 was reduced in the presence of longer FAs [81]. Further, α-secretase activity as well as IDE-dependent Aβ-degradation are stimulated by FAs consisting of 10 to 14 carbon atoms. Both these effects would likely lead to reduced Aβ levels [71,82]. In line with this assumption, a trend towards decreased total Aβ levels in the brain tissue of dogs receiving MCTs for two months has been reported. However, this tendency was traced to reduced steady-state levels of the precursor APP [83]. In this context, a recent publication by Shippy et al. should be mentioned that reported the MCFA metabolite and ketone body BHB to reduce overall AD pathology in an AD mouse model. In this study, exogenous BHB administration reduced amyloid plaque formation, microgliosis, and caspase-1 activation by inhibiting the NLRP3 inflammasome activation. The NLRP3 inflammasome is known to control caspase-1 activation and the release of interleukins (IL-1β and IL-18) in macrophages [84]. In strong analogy to ω-3-PUFAs, pure MCFAs, coconut oil, and ketones have been additionally demonstrated to reduce the susceptibility of primary neurons to Aβ-induced toxicity, further emphasizing the neuroprotective potential of these natural compounds [85,86,87].

## 5. The Impact of ω-3-PUFAs and MCFAs on Oxidative Stress 

One of the molecular mechanisms underlying Aβ-induced toxicity is the induction of oxidative stress, which is defined as ‘an imbalance in pro-oxidants and antioxidants with associated disruption of redox circuitry and macromolecular damage‘ [88]. It is characterized by increased levels of reactive oxygen and nitrogen species (ROS, RNS) such as O_2_^•−^ (superoxide radical anion), OH^•^ (hydroxyl radical), H_2_O_2_ (hydrogen peroxide), ^•^NO (nitric oxide), and ONOO^−^ (peroxynitrite). The major cellular sources of these highly reactive molecules are the mitochondrial electron transport chain and the family of NADPH oxidases (NOXs) mainly producing O_2_^•−^ and H_2_O_2_. There are two different kinds of ROS-detoxifying defense mechanisms within brain tissue: 1. The antioxidant enzyme system and 2. low-molecular weight antioxidants. The antioxidant enzyme system includes superoxide dismutase (SOD), glutathione reductase (GR), glutathione peroxidase (GPx), and catalase (CAT). O_2_^•−^ is rapidly dismutated by SODs to H_2_O_2_, which produces the highly reactive OH^•^ if it is not detoxified by CAT and GPx. Low molecular weight antioxidants such as glutathione, uric acid, and ascorbic acid neutralize ROS by causing the chelation of transition metals [89,90,91]. In moderate or low amounts, ROS play a key role as messengers in normal cell signal transduction, whereas in excess they are hazardous. They can oxidize all major biomolecules (nucleic acids, proteins, and lipids) preventing them from performing their original functions. By affecting phospholipid asymmetry in cellular membranes, lipid peroxidation could be responsible for a disruption of membrane integrity, for example [92]. Accordingly, such oxidative modifications can finally lead to cellular degeneration, functional decline, and cell death [89,93,94]. Because of its high oxygen consumption rate, its high content of PUFAs, and its relatively poor antioxidative mechanisms, the human brain is extremely susceptible to ROS insults [95]. Several markers of oxidative stress such as lipid peroxidation products (4-hydroxy-2-nonenal (HNE), F2-isoprostanes, F4-isoprostanes), and oxidatively-modified proteins are significantly enhanced in the brain tissue and cerebrospinal fluid (CSF) of MCI- and AD-patients [92,96,97,98,99,100,101,102]. This clearly points to increased oxidative injuries under the pathological conditions of AD. 

Despite the high oxidizability of ω-3-PUFAs, their mere presence in tissues does not predispose the membranes to oxidative stress. As shown in animal models, dietary supplementation of ω-3-PUFAs rather ameliorates oxidative insults within brain tissue. Chronic administration of DHA (22:6ω-3) to Aβ40-infused rats suppressed the cortical and hippocampal increase in lipid peroxide and ROS levels. Further, the incorporation of DHA (22:6ω-3) and EPA (20:5ω-3) into the brain tissue of aged Wistar rats reduced lipid peroxidation and increased SOD activity [103,104]. In another study, the oral administration of DHA (22:6ω-3) stimulated the antioxidant defenses by enhancing CAT- and GPx-activity as well as the level of glutathione in rat cerebrum. This might be based on alterations in the enzymes’ gene expression as demonstrated for phospholipid-hydroperoxide glutathione peroxidase (Gpx4) in the murine hippocampus and for CAT and GPx in hepatic tissues and rat skeletal muscle [33,105,106,107]. ω-3-PUFAs and their oxidation products can facilitate the antioxidative defense by raising the activity of the nuclear erythroid factor-2 (Nrf2). This stress-responsive transcription factor mediates the coordinated regulation of multiple antioxidant genes including those encoding for SOD1 and SOD2, CAT, and GPx by binding to antioxidant response elements (AREs) in their promoter regions [108]. As demonstrated in AD model mice, the induction of Nrf2 prevents cognitive impairment by suppressing oxidative stress and neuroinflammation, suggesting that Nrf2 might be an important therapeutic target regarding AD [109]. In a recent study, a direct link between the DHA (22:6ω-3)-dependent activation of Nrf2 signaling pathways and the reduction of oxidative damage that was caused by Aβ25–35 was shown in PC12 cells [110]. In this context it should be mentioned that there are many bioactive metabolites of ω-3-PUFAs such as protectins, resolvins, and maresins, which might mediate some of their antioxidative and anti-inflammatory effects [2,33].

Less is known about the impact of dietary MCFAs on oxidative stress parameters. The MCFA lauric acid (12:0) has been recently reported to induce myocardial oxidative stress and atrophy in mice [111]. In contrast, in human liver cells MCFAs evoked lower levels of the oxidative stress marker malondialdehyde (MDA) and lessened inflammation and apoptosis compared to LCFAs [112,113]. As already mentioned above, beneficial effects of MCFA have been also described in neuronal cells. MCFAs protected cortical neurons from Aβ-induced toxicity and coconut oil diminished oxidative stress markers in these cells [86,87]. In SH-SY5Y cells that were exposed to decanoic acid (10:0) a marked increase in mitochondrial citrate synthase and complex I activity has been shown by Hughes et al., indicating an elevated mitochondrial number which might affect O_2_^•−^ production. The authors propose that this effect might be the consequence of the activation of PPARγ and hence the altered expression of target genes that are involved in mitochondrial biogenesis [114]. PPARγ belongs to the PPAR family of ligand-activated nuclear receptors acting as transcription factors. They are known to be activated by different FAs including ω-3-PUFAs and MCFAs, and other endogenous lipidic compounds. After ligand binding, PPARs form a heterodimer with the retinoid X receptor (RXR) and recruit co-activators. The complex then binds to specific regions on the deoxyribonucleic acid (DNA) of target genes called peroxisome proliferator response elements (PPREs) and regulate gene transcription [14,115,116,117]. In addition, the authors found a stimulated CAT activity in these cells, while the cellular content of reduced glutathione (GSH) was unaffected [114]. We recently also observed an improved antioxidative status along with reduced ROS levels in neuroblastoma cells that were supplemented with phosphatidylcholine (PC) containing decanoic acid (PC10:0/10:0) [68]. The deepened examination revealed PC10:0/10:0 to reduce the cellular H_2_O_2_ release by elevating CAT activity and hence H_2_O_2_ detoxification. However, in contrast to the study by Hughes et al., this effect was independent of the transcription factor PPARγ and of alterations in CAT gene expression in our study. Accordingly, the CAT-stimulating effect of PC10:0/10:0 might be based on the direct effects on the enzyme’s catalytic activity protecting the cells from H_2_O_2_ [68], a key player in Aβ-induced toxicity [118]. It should be noted that in both studies the observed effects of decanoic acid (10:0) seemed not to be mediated by ketone bodies, but rather directly by the FA [68,114]. As reported by others, MCFAs also reduced the H_2_O_2_ production in mouse skeletal muscle and lessened ROS levels in C2C12 myotubes compared to LCFAs [119]. 

## 6. The Impact of ω-3-PUFAs and MCFAs on Neuronal Energy Metabolism

Neurons are high energy-demanding cells which need to be constantly supplied with oxygen and energy for maintaining ionic gradients and sustaining their functions. The major energy source of these cells is glucose which is metabolized to adenosine triphosphate (ATP) via glycolysis, the tricarboxylic acid (TCA) cycle, and the electron transport chain. Glucose enters the brain from the vasculature and is taken up by neurons predominantly through the highly efficient glucose transporters 1 and 3 (GLUT1, GLUT3), respectively [120,121]. A dysfunctional cerebral glucose utilization, secondary to oxidative stress, is considered as a key event in AD pathogenesis. A reduced neuronal uptake and metabolism of glucose leads to inefficient glycolysis and is finally closely linked to progressive cognitive decline. Already decades prior to the clinical manifestation of AD, a deterioration in brain energy metabolism specific to glucose can be measured by Positron emission tomography (PET) imaging [11,122,123,124,125,126]. This could be due to the conjugation of lipid peroxidation products with proteins that are involved in energy metabolism. Oxidation-induced alterations in the structure and function of transporters and enzymes that are involved in ATP production and glucose metabolism (e.g., ATP synthase, aldolase, triose phosphate isomerase, aconitase, and pyruvate kinase) seem to be associated with AD pathology. Additionally, a reduced neuronal expression of genes encoding for subunits of the mitochondrial electron transport chain has been reported. The resulting decreased cellular ATP levels can cause electron leakage and mitochondrial ROS overproduction. Thus, mitochondria are considered both as major target of oxidative damage and as one of the most important sources of ROS generation [32,126,127,128,129,130,131]

ω-3-PUFAs have been demonstrated to propagate their neuroprotective effects by improving mitochondrial function and cerebral glucose metabolism. Fish oil restored the age-related decrease in respiration and improved ATP production in the brain of aged mice [132]. DHA (22:6ω-3) has been shown to diminish a hyperoxia-induced surge in mitochondrial ROS production, to preserve mitochondrial Ca^2+^ buffering capacity, and to improve mitochondrial dynamics in vitro and in vivo [133,134,135]. A body of evidence suggests that dietary ω-3-PUFAs might also have important functions in the regulation of brain glucose utilization. ω-3-PUFA-deficient animals show an impaired neurotransmission, which is probably closely linked to a disturbed brain glucose utilization. In contrast, ω-3-PUFA-supplementation results in a higher brain glucose uptake along with an increased cerebral metabolic rate of glucose [136,137,138,139]. These effects seem to be based on a ω-3-PUFA-dependent modulation of the glucose transport in endothelial cells of the blood-brain barrier. The FAs probably affect the protein amount and activity of GLUT1 at the post-transcriptional and/or post-translational level [138,140,141]. In addition, in ω-3-PUFA-deprived rats, a decreased respiratory chain activity was found as indicated by a reduced cytochrome oxidase (CO) activity. In line with this, a stimulating effect of dietary ω-3-PUFAs on the expression of genes encoding for enzymes that are involved in energy metabolism such as CO, NADH dehydrogenase, and ATP synthase in rat brains has been reported [142,143].

In contrast to LCFAs, MCFAs are directly absorbed from the gut into the portal vein and do not require a carnitine-dependent activation before accessing the mitochondria where they are used as substrates in mitochondrial β-oxidation and the TCA cycle. Moreover, the cellular uptake and transport of MCFAs is independent of FA transport proteins such as plasma membrane-embedded FA translocase and cytosolic FABPs [10,144]. The consequence is an increased hepatic metabolization of MCFAs into ketone bodies. After their monocarboxylic acid transporter 1 (MCT1)-dependent transport across the blood brain-barrier, these hepatically derived ketone bodies can be used as additional fuel by the neuronal cells [4,11,67,145]. In strong contrast to glucose utilization, the metabolism of ketone bodies is unaltered in AD, at least in the early stages of the disease. Thus, MCFA-derived ketone bodies might serve as an alternative fuel in the brain of AD patients compensating for the lack of cerebral glucose utilization [1]. For this reason, the beneficial effects of MCFAs in helping ameliorate the cognitive decline that is caused by AD are generally attributed to the resulting ketones boosting brain energy supply [4,11,67]. However, MCFAs also have cognition- and synaptic plasticity-enhancing properties in rats which are not related to ketone production [66]. As already mentioned above, MCFAs and, in particular, decanoic acid (10:0), seem to alter cell energetics by enhancing mitochondrial function. PPARγ activation by decanoic acid (10:0) has been shown to trigger mitochondrial biogenesis and to prevent glucose deprivation-induced neuronal death. The resulting higher number of mitochondria in neuroblastoma cells that are supplemented with decanoic acid (10:0) results in an elevated TCA cycle and mitochondrial complex I activity and finally in an increased ATP availability [11,114,117,146]. The FA has been also reported to increase mitochondrial function and ATP synthesis capacity in vivo in a mouse model [147].

## 7. Conclusions

**Figure 4:**
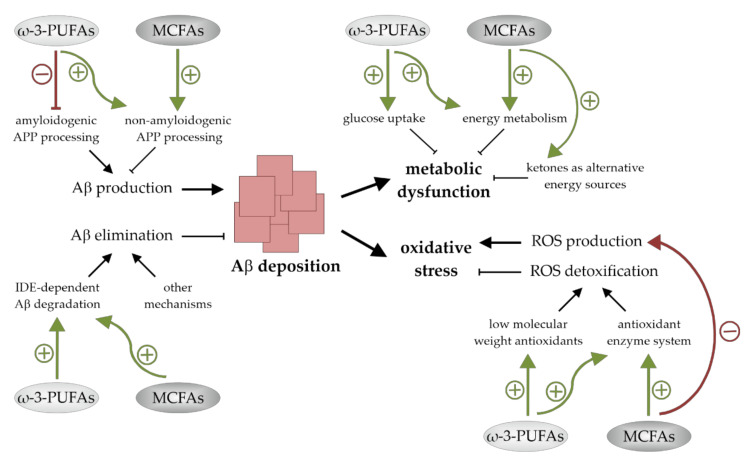
Summary of the mechanisms by which ω-3-polyunsaturated fatty acids (ω-3-PUFAs) and medium chain fatty acids (MCFAs) reduce cerebral amyloid-β (Aβ) levels, improve brain energy metabolism, and lessen oxidative stress. Both ω-3-PUFAs and MCFAs reduce Aβ deposition by stimulating non-amyloidogenic amyloid precursor protein (APP)-processing and insulin-degrading enzyme (IDE)-dependent Aβ degradation. ω-3-PUFAs additionally suppress Aβ production. Metabolic dysfunction and oxidative stress are also lessened by both fatty acid (FA) classes. Glucose uptake and energy metabolism are increased by ω-3-PUFAs, while oxidative stress levels are reduced in the presence of these FAs due to an increased ROS detoxification. MCFAs improve the cellular metabolic function as well and lower oxidative stress by stimulating reactive oxygen species (ROS) detoxification and reducing ROS generation.

AD, the most common cause of dementia in the elderly population, is associated with an altered cerebral lipid homeostasis and changes in brain FA composition. A body of evidence supports the fact that the dietary supplementation of ω-3-PUFAs and MCFAs could be valuable for disease modification. They have neuronal health promoting and cognition-enhancing properties and might be of benefit for humans that are affected by MCI and early stages of AD. On the cellular level there are indications that these FAs promote their neuroprotective actions via additive pleiotropic mechanisms. These include effects on Aβ accumulation as well as on mechanisms that are involved in Aβ-induced neuro- and synaptotoxicity. Both ω-3-PUFAs and MCFAs seem to reduce cerebral Aβ deposition, to lower oxidative stress levels, and to positively affect brain energy metabolism (Figure 4). A deeper understanding of the molecular mechanisms by which these bioactive compounds exert their beneficial effects could lead to further progression in the development of nutritional therapies for AD and maybe other currently incurable nervous system disorders.

## Figures and Tables

**Table 1:** Overview of most typical fatty acids.

Class	Common Name	C:D	ω−x	Chemical Formula
Saturated Fatty Acids (SFAs)				
SCFAs	Butyric acid	4:0		CH_3(_CH_2)2_COOH
	Caproic acid	6:0		CH_3_(CH_2_)_4_COOH
MCFAs	Caprylic acid	8:0		CH_3_(CH_2_)_6_COOH
	Decanoic acid (Capric acid)	10:0		CH_3_(CH_2_)_8_COOH
	Lauric acid	12:0		CH_3_(CH_2_)_10_COOH
LCFAs	Myristic acid	14:0		CH_3_(CH_2_)_12_COOH
	Palmitic acid	16:0		CH_3_(CH_2_)_14_COOH
	Stearic acid	18:0		CH_3_(CH_2_)_16_COOH
VLCFAs	Arachidic acid	20:0		CH_3_(CH_2_)_18_COOH
	Behenic acid	22:0		CH_3_(CH_2_)_20_COOH
	Lignoceric acid	24:0		CH_3_(CH_2_)_22_COOH
Monounsaturated fatty acids (MUFAs)				
MUFAs	Palmitoleic acid	16:1	ω-7	CH_3_(CH_2_)_5_CH=CH(CH_2_)_7_COOH
	Oleic acid	18:1	ω-9	CH_3_(CH_2_)_7_CH=CH(CH_2_)_7_COOH
	Erucic acid	22:1	ω-9	CH_3_(CH_2_)_7_CH=CH(CH_2_)_11_COOH
Polyunsaturated fatty acids (PUFAs)				
ω3-PUFAs	α-Linolenic acid (ALA)	18:3	ω-3	CH_3_CH_2_CH=CHCH_2_CH=CHCH_2_CH=CH(CH_2_)_7_COOH
	Eicosapentaenoic acid (EPA)	20:5	ω-3	CH_3_CH_2_CH=CHCH_2_CH=CHCH_2_CH=CHCH_2_CH=CHCH_2_CH=CH(CH_2_)_3_COOH
	Docosahexaenoic acid (DHA)	22:6	ω-3	CH_3_CH_2_CH=CHCH_2_CH=CHCH_2_CH=CHCH_2_CH=CHCH_2_CH=CHCH_2_CH=CH(CH_2_)_2_COOH
ω6-PUFAs	Linoleic acid (LA)	18:2	ω-6	CH_3_(CH_2_)_4_CH=CHCH_2_CH=CH(CH_2_)_7_COOH
	Arachidonic acid (AA)	20:4	ω-6	CH_3_(CH_2_)_4_CH=CHCH_2_CH=CHCH_2_CH=CHCH_2_CH=CH(CH_2_)_3_COOH

C:D: total amount of carbon (C) atoms of the fatty acid, and the number of double (D) bonds in it; ω−x (also n-x): position of the first double bond counting from the terminal methyl carbon (referred to as n or ω) toward the carbonyl carbon; SFAs: saturated fatty acids; SCFAs: short chain fatty acids; MCFAs: medium chain fatty acids; LCFAs: long chain fatty acids; VLCFAs: very long chain fatty acids; MUFAs: monounsaturated fatty acids; PUFAs: polyunsaturated fatty acids.
